# Hypohydration induced by prolonged cycling in the heat increases biomarkers of renal injury in males

**DOI:** 10.1007/s00421-023-05328-8

**Published:** 2023-10-17

**Authors:** Loris A. Juett, Jack E. Drury, Thomas B. Greensmith, Alfie P. Thompson, Mark P. Funnell, Lewis J. James, Stephen A. Mears

**Affiliations:** 1 School of Sport, Exercise and Health Sciences, Loughborough University, Leicestershire, LE11 3TU UK; 2 Loughborough College, Loughborough, LE11 3BT UK

**Keywords:** Dehydration, Water, NGAL, KIM-1, AKI, Exercise

## Abstract

**Purpose:**

Recent studies have shown that hypohydration can increase renal injury. However, the contribution of hypohydration to the extent of renal injury is often confounded by exercise induced muscle damage. Therefore, the aim of the present study was to investigate the effect of manipulating hydration status during moderate-intensity cycling in the heat on biomarkers of renal injury.

**Methods:**

Following familiarisation, fourteen active males (age: 21 [20–22] y; BMI: 22.1 ± 1.9 kg/m^2^; $\dot{V}$O_2peak_: 55 ± 9 mL/kg/min) completed two experimental trials, in a randomised cross-over design. Experimental trials consisted of up to 120 min of intermittent cycling (~ 50% W_peak_) in the heat (~ 35 °C, ~ 50% relative humidity). During exercise, subjects consumed either a water volume equal to 100% body mass losses (EU) or minimal water (HYP; 75–100 mL) to induce ~ 3% body mass loss. Blood and urine samples were collected at baseline, 30 min post-exercise and 24 h post-baseline, with an additional urine sample collected immediately post-exercise.

**Results:**

Thirty minutes post-exercise, body mass and plasma volume were lower in HYP than EU (*P* < 0.001), whereas serum and urine osmolality (*P* < 0.001), osmolality-corrected urinary kidney injury molecule-1 concentrations (HYP: 2.74 [1.87–5.44] ng/mOsm, EU: 1.15 [0.84–2.37] ng/mOsm; *P* = 0.024), and percentage change in osmolality-corrected urinary neutrophil gelatinase-associated lipocalin concentrations (HYP: 61 [17–141] %, EU: 7.1 [– 4 to 24] %; *P* = 0.033) were greater in HYP than EU.

**Conclusion:**

Hypohydration produced by cycling in the heat increased renal tubular injury, compared to maintaining euhydration with water ingestion.

## Introduction

Acute kidney injury (AKI) is relatively common following prolonged endurance events, but this diagnosis typically derives from alterations in serum creatinine concentrations (a product of muscle breakdown that is filtered and excreted by the kidneys) (Hodgson et al. 2017; Juett et al. 2021a). Therefore, it can be unclear to what extent this post-exercise AKI reflects genuine renal injury, rather than increased muscle breakdown and/or a benign reduction in renal blood flow due to increased skeletal muscle and skin blood flow (Rowell 1974; Poortmans 1984; Hoffman and Weiss 2016; Hodgson et al. 2017). Consequently, recent work has examined the responses of biomarkers that are more indicative of renal tubular injury (Kashani et al. 2017; Mansour et al. 2017; Schlader et al. 2019), such as urinary neutrophil gelatinase-associated lipocalin (uNGAL) and urinary kidney injury molecule-1 (uKIM-1), showing increases in the concentrations of these biomarkers following exercise (Juett et al. 2021a).

Factors that likely contribute to exercise-associated increases in renal tubular injury include muscle damage, increases in sympathetic tone and body temperature, and hypohydration (Poortmans 1984; Junglee et al. 2013; Hoffman and Weiss 2016). Hypohydration is of particular interest because it is relatively simple to minimise with fluid consumption and has the potential to attenuate exercise-associated renal injury via several mechanisms (Smith et al. 1952; Bragadottir et al. 2009; Juett et al. 2021a). Recently, we showed that the maintenance of euhydration (through water ingestion) during high intensity intermittent running in temperate conditions attenuated the post-exercise increase in osmolality-corrected uKIM-1 concentrations, compared to when fluid was restricted and hypohydration accrued, suggesting that euhydration attenuated injury to the proximal tubules (Juett et al. 2021b). Similarly, Chapman et al. (2020) also reported that maintaining euhydration with water ingestion attenuated the increase in a marker of proximal tubular injury (urinary insulin-like growth factor-binding protein 7; uIGFBP7) following treadmill walking in the heat (Chapman et al. 2020).

Whilst the findings from these studies suggest that maintaining euhydration with water ingestion during exercise attenuates proximal tubular injury (Chapman et al. 2020; Juett et al. 2021b), these studies have focussed on walking/running, where the eccentric components of exercise may produce muscle damage that may exacerbate renal injury (Junglee et al. 2013). The predominantly concentric nature of the muscle contractions when cycling produces minimal muscle damage (Peñailillo et al. 2013), allowing any potential effects of manipulating hydration status on renal injury to be further isolated. Furthermore, despite being able to carry a reasonable amount of fluid, hypohydration is common in cyclists as intake often fails to meet sweat losses (Atkinson et al. 2003), meaning that understanding the influence of different hydration interventions is also of practical importance. Additionally, cycling is commonly performed in the heat, which, in addition to the increased sweat loss, may also increase the risk of renal injury due to a greater demand for skin blood flow (to dissipate heat), reducing renal blood flow and increasing the risk of ischaemia and subsequent renal injury (Radigan and Robinson 1949; Smith et al. 1952; Rowell 1974; Basile et al. 2012; Sato et al. 2019). If manipulating hydration status during cycling in the heat affects biomarkers of renal injury, this could have implications for individuals that regularly partake in this form of exercise.

Therefore, the aim of the present study was to investigate the effect of manipulating hydration status during cycling in the heat on biomarkers of renal injury. It was hypothesized that hypohydration produced by cycling in the heat would result in greater post-exercise concentrations of uKIM-1, compared to when euhydration was maintained with water ingestion.

## Methods

### Subjects

Fourteen active males (age: 21 [20–22] y; height: 1.82 ± 0.08 m; body mass: 73.0 ± 9.6 kg; BMI: 22.1 ± 1.9 kg/m^2^; $\dot{V}$O_2peak_: 55 ± 9 mL/kg/min; W_peak_: 295 ± 42 W), who were non-smokers and had no known history of kidney issues, completed this study. Exclusion criteria included regular use of medications (e.g., non-steroidal anti-inflammatory drugs), or any current medical complications, which could influence kidney function. This study was performed in line with the principles of the Declaration of Helsinki. Ethical approval was granted from the Loughborough University Ethics Approvals (Human Participants) Sub-Committee.

### Study design

Subjects visited the laboratory on five occasions: preliminary visit and two experimental trials (two visits per experimental trial). Experimental trials were completed in a randomised, counter-balanced order and involved intermittent cycling in the heat (~ 35 ˚C) with either water ingestion to replace body mass losses (EU) or minimal water ingestion (75–100 mL; HYP), followed by a ~ 20.5 h recovery period. Trials were separated by ≥ 7 days. Data collection occurred from October 2019 to March 2020.

### Preliminary visit

Subjects completed a health screen questionnaire, provided verbal and written informed consent, and were familiarised to blood sampling procedures. Height and nude body mass (AFW-120 K, Adam Equipment Co., UK) were measured, followed by determination of peak oxygen uptake ($\dot{V}$˙O_2peak_) and peak power output (W_peak_) using a cycle ergometer (Lode Corival, Groningen, the Netherlands). Subjects cycled at 95 W in a temperate environment (~ 21 °C), with power output increasing by 35 W every 3 min, until volitional exhaustion. A final ~ 60 s expired gas sample was collected into a Douglas bag and analyzed for O_2_ and CO_2_ concentrations (Servomex 1400 Gas Analyzer, Servomex, Crowborough, UK), temperature (RS Pro Digital Thermometer, RS components, Corby, UK) and volume (Harvard Dry Gas 194 Meter, Harvard Apparatus Ltd, Edenbridge, UK). Ambient air was simultaneously collected to correct $\dot{V}$O_2_ and $\dot{V}$CO_2_ values (Betts and Thompson 2012). After sufficient rest, subjects entered an environmental chamber (~ 35˚C, ~ 50% relative humidity) and cycled for 30 min at ~ 50% W_peak_. During this time, heart rate (HR), rating of perceived exertion (RPE; 6–20), thermal sensation (scale of – 10 = extremely cold, to 10 = extremely hot) and aural temperature (Braun ThermoScan 3, ear thermometer, Lausanne, Switzerland) were recorded. Pre- and post-exercise nude body mass measurements, as well as the volume of water consumed ad libitum, were used to estimate sweat losses and calculate fluid replacement for the first 20 min of the EU trial.

### Pre-trial standardisation

The day before their first experimental trial, subjects recorded their food and fluid intake, ensuring consumption of ≥ 40 mL/kg body mass of fluid (compliance aided by marked bottles), and refrained from strenuous exercise and alcohol consumption. This was replicated prior to the second experimental trial. Subjects were sent reminders regarding pre-trial standardisation two days prior to experimental trials, with compliance verbally checked upon arrival for experimental trials.

### Experimental trials

Subjects reported to the laboratory between 7:30 and 9:00am (time standardised within subject; after ≥ 10 h overnight fast) and commenced seated rest. During which, subjects completed a series of subjective feelings questionnaires (headache, nausea, dizziness, thirst, thermal sensation, GI comfort, stomach fullness, GI bloating and urge to vomit), which were all on a scale of 0 (no symptom) to 10 (maximum symptom), except thermal sensation. Aural temperature was also measured. After 30 min, a blood sample was taken, a urine sample provided, and nude body mass was measured. Over 15 min, subjects consumed a standardised breakfast of cereal biscuits (Belvita, Mondelez, Uxbridge, UK/ Nature Valley, General Mills, Uxbridge, UK) and 4 mL/kg body mass of a glucose-based sport drink (Lucozade Sport, Lucozade Ribena Suntory Limited, Uxbridge, UK), providing 1 g carbohydrate/kg body mass in total. Subjects then entered the environmental chamber (~ 35 °C; ~ 50% relative humidity), nude body mass was measured, and cycling commenced. The cycling period consisted of 30 min cycling at ~ 50% W_peak_, followed by a nude body mass measurement, and then a repeated sequence of ~ 12 min of cycling, followed by ~ 3 min rest. Nude body mass measurements were made during the ~ 3 min rest periods. Subjects cycled until 120 min had elapsed or until nude body mass had decreased by > 3% compared to their post-breakfast mass (HYP)/ until they had consumed a volume of water > 3% of their post-breakfast mass (EU). Exercise time was matched within subject, based on the first trial completed. In EU, subjects consumed water equal to 33% of their 30 min sweat losses (calculated from the preliminary trial) at 10 and 20 min. After this, body mass losses (compared to post-breakfast body mass) were 100% replaced with water ingestion after each nude body mass measurement. In HYP, to reduce unpleasant mouth dryness, subjects received 25 mL water every 30 min. All water provided to subjects during exercise had been stored in bottles in a water bath maintained at ~ 37˚C. Every 15 min, HR, RPE and aural temperature (data not presented, but collected for safety requirements) were recorded. Environmental temperature and relative humidity (Kestrel 4400, Nielsen-Kellerman Co, Boothwyn, USA) were recorded throughout exercise. If subjects found it difficult to complete a block of exercise, or if aural temperature reached 38.4˚C, cycling power output was decreased; this was repeated at the same time in the subsequent trial. If aural temperature exceeded 38.5˚C, the trial was terminated and the subject was withdrawn from the study (*n* = 2). Subjective feelings questionnaires were completed every 30 min during exercise. Upon finishing exercise, subjects provided a urine sample, had nude body mass measured, then commenced seated rest. In EU, subjects had 10 min to consume a water volume equal to any remaining body mass losses. In HYP, subjects consumed 25 mL water. Subjects completed the subjective feelings questionnaires, before a blood sample was taken after 30 min. After this, another urine sample was collected, and nude body mass was measured. Subjects then left the laboratory and recorded ad libitum food and fluid intake for the remainder of the day, using food weighing scales (Salter ARC digital kitchen scale, Manchester, UK) and a diet diary, which was later analyzed using online software (Nutritics, Dublin, Ireland). Subjects collected all urine produced until returning to the laboratory the following morning (24 h post-baseline), after an overnight fast (≥ 10 h), to complete subjective feelings questionnaires, have blood and urine samples collected, and nude body mass measured.

### Sample analysis

All blood samples were taken by venepuncture of an antecubital vein. From each blood sample, 1 mL was dispensed into a tube containing K_2_EDTA (1.75 mg/L, Teklab, Durham, UK) and was used to measure haematocrit (microcentrifugation; Hawksley Microhematocrit Centrifuge, Hawksley, Worthing, UK) and haemoglobin concentration (cyanmethaemoglobin method), to estimate plasma volume changes from baseline (Dill and Costill 1974). Another 4.5 mL blood was dispensed into a tube containing a clotting catalyst (Sarstedt Ltd, Leicester, UK), which was centrifuged (2200 g, 15 min, 4 °C) after at least 20 min at room temperature. Serum was aliquoted and stored at − 80 °C. Urine samples were measured for osmolality (Osmocheck; Vitech Scientific, Horsham, UK) and were then aliquoted and stored at − 80 °C.

A bench-top analyser (ABX Pentra C400; Horiba medical, Northampton, UK) was used to measure creatine kinase (CK), creatinine, myoglobin, and uric acid concentrations in serum. The intra-assay CV for serum CK, creatinine, myoglobin and uric acid concentrations were 0.7, 2.3, 3.9 and 0.7%, respectively. Freezing-point depression (Osmomat Auto, Cryoscopic Osmometer, Gonotec, Berlin, Germany) was used to measure serum osmolality. ELISA kits were used to determine uKIM-1 (KIM-1 Human ELISA Kit, Enzo Life Sciences, Lausen, Switzerland) and uNGAL (Human NGAL ELISA Kit, BioPorto, Hellerup, Denmark) concentrations. The intra-assay CV for uKIM-1 and uNGAL concentrations were 3.5 and 4.1%, respectively.

### Data and statistical analyses

Urine biomarkers were corrected for urine osmolality, and relevant serum markers were corrected for changes in plasma volume. All blood markers are *n* = 13 because blood samples were not collected from one subject. Body mass and subjective feelings questionnaires are *n* = 13 and *n* = 12, respectively, due to issues during data collection. Subjective feelings questionnaires are presented as: baseline, 30 min, 60 min, end of exercise, post-exercise and 24 h post-baseline (to account for the endpoint of exercise finishing before 120 min in three subjects). As the manipulation of water intake (10 min into exercise) had begun before the first HR and RPE measurements (15 min into exercise) were taken, these measures were averaged for each trial. Food and fluid intakes are presented as *n* = 12 because two subjects’ diet diaries were not provided in sufficient detail to accurately analyse. If a subject was unable to produce a urine sample either immediately post-exercise (*n* = 1) or 30 min post-exercise (*n* = 2), then the urine produced at the other timepoint (i.e., immediately post-exercise or 30 min post-exercise) was assumed to be both their immediately and 30 min post-exercise urine samples.

SPSS (version 27, SPSS, Armonk, NY, USA) was used to perform statistical analyses. Shapiro–Wilk tests were used to check if data followed a Gaussian distribution. Data with one factor (trial), such as ad libitum food and fluid intake, HR and RPE, were compared using a paired-samples *t* test or Wilcoxon signed-rank test, depending on a Gaussian distribution. Data containing two factors (trial and time), were analyzed using a two-way repeated measures ANOVA. If the assumption of sphericity was violated, the Greenhouse–Geisser correction was used. Holm-Bonferroni adjusted post hoc paired-samples *t*-tests or Wilcoxon signed-rank tests (as appropriate) were used to further investigate significant ANOVA effects. Data that follow a Gaussian distribution are displayed as (mean ± SD), whereas data that do not follow a Gaussian distribution are displayed as (median [interquartile range]). Effect sizes (partial eta squared) where calculated for trial x time interactions, where small, medium and large effects were defined as 0.01, 0.06 and 0.14 respectively (Cohen 1988). Power calculations performed in previous studies indicated that a sample size of 6–12 subjects would be required to detect the smallest relevant change in uNGAL concentrations between trials, with an alpha of 0.05 and a statistical power of 0.8 (Junglee et al. 2013; Chapman et al. 2020). In line with the sample size of prior studies that have reported differences in biomarkers of renal tubular injury between hypohydrated and euhydrated trials (Chapman et al. 2020; Juett et al. 2021b), the present study achieved a sample size of fourteen subjects.

## Results

### Trial conditions

There were no baseline differences between trials for baseline body mass (HYP: 73.6 ± 9.4 kg, EU: 73.7 ± 9.2 kg; *P* = 0.693), serum (HYP: 291 ± 2 mOsm/kgH_2_O, EU: 291 ± 3 mOsm/kgH_2_O; *P* > 0.999) and urine (HYP: 670 ± 160 mOsm/kgH_2_O, EU: 720 ± 150 mOsm/kgH_2_O; *P* = 0.579) osmolality, haemoglobin (HYP: 15.7 ± 0.8 g/dL, EU: 15.9 ± 0.8 g/dL; *P* = 0.107), haematocrit (HYP: 43.4 ± 1.7%, EU: 43.7 ± 1.5%; *P* = 0.423) and thirst sensation (HYP: 4 [3–5]; EU: 4 [2–5]; *P* = 0.788), indicating subjects arrived at the laboratory in a similar hydration state for both trials. During exercise, ambient temperature (HYP: 35.3 [35.2–35.3] °C; EU: 35.2 [35.2–35.3] °C; *P* = 0.380) and relative humidity (HYP: 54.4 ± 0.9%; EU: 54.7 ± 0.9%; *P* = 0.338) were not different between trials.

### Hydration status measurements

**Fig. 1:**
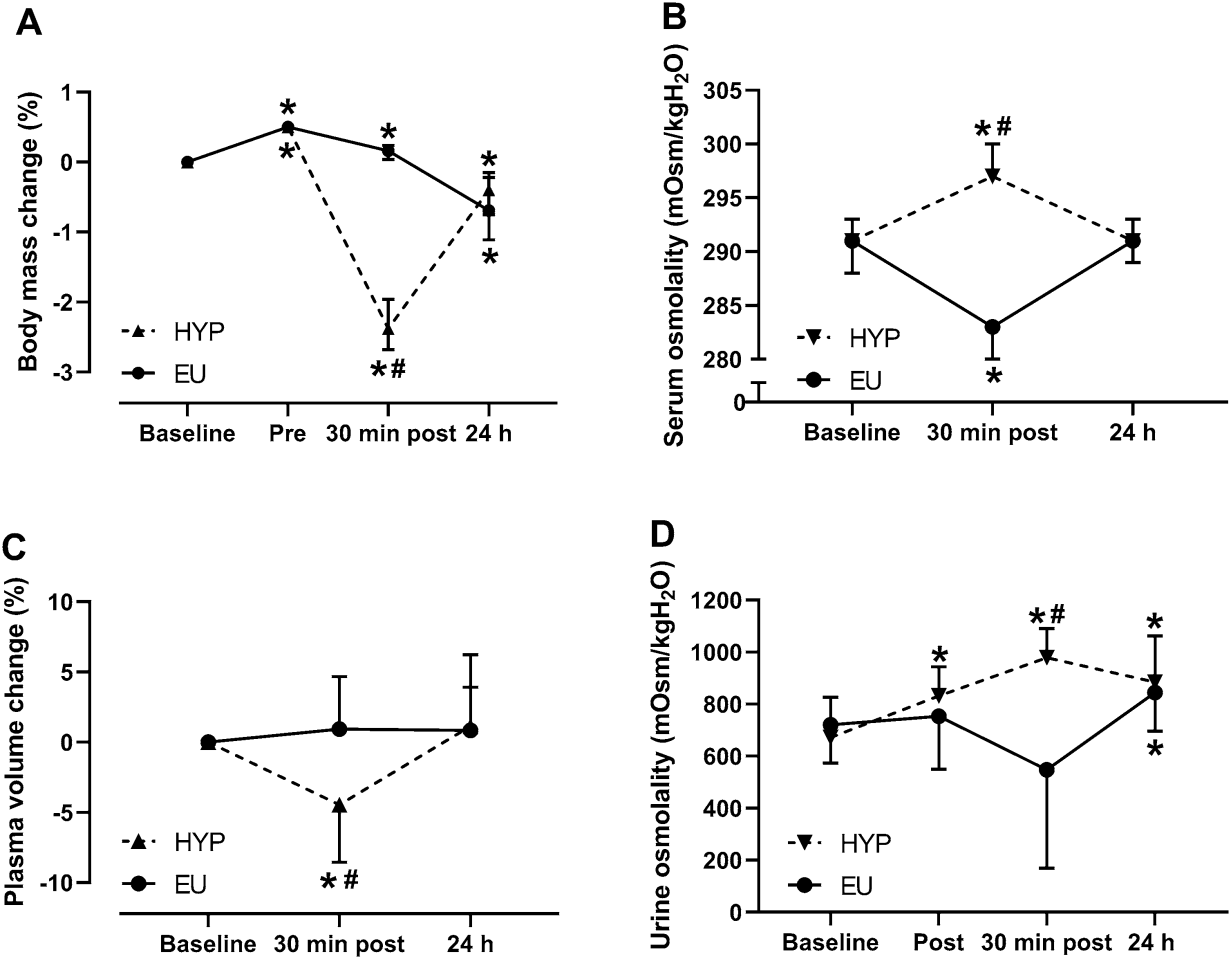
Change in body mass relative to baseline (**A**), serum osmolality (**B**), change in plasma volume relative to baseline (**C**) and urine osmolality (**D**). Timepoints include: baseline, immediately before exercise (Pre), immediately after exercise (Post), 30 min after exercise (30 min post) and 24 h post-baseline (24 h). *Indicates significantly different from baseline; ^#^Indicates significant difference between Hypohydrated (HYP) and Euhydrated (EU) trials. Data in (**A**) are presented as median with interquartile range. Data in (**B**–**D**) are presented as mean ± standard deviation

There were trial by time interaction effects (*P* ≤ 0.004) for changes in body mass ($\eta_p^2$= 0.942; Fig. 1A) and plasma volume ($\eta_p^2$= 0.484; Fig. 1C), as well as serum ($\eta_p^2$= 0.933; Fig. 1B) and urine ($\eta_p^2$= 0.547; Fig. 1D) osmolality. Thirty minutes post-exercise, body mass and plasma volume were lower in HYP than EU (*P* < 0.001), whereas serum and urine osmolality were higher in HYP than EU (*P* < 0.001). In HYP, body mass decreased from baseline to 30 min post-exercise (*P* < 0.001), whereas in EU, body mass increased (*P* = 0.009), mainly due to the ingestion of the pre-exercise breakfast. At 24 h post-baseline, body mass was lower compared to baseline in both trials (*P* ≤ 0.016), but not different between trials (*P* = 0.964). Plasma volume decreased from baseline to post-exercise in HYP (*P* = 0.004) but did not change in EU (*P* = 0.772). At 24 h post-baseline, plasma volume was not different to baseline in either trial (*P* ≥ 0.169). Serum osmolality increased from baseline to 30 min post-exercise in HYP (*P* < 0.001) but decreased in EU (*P* < 0.001). At 24 h post-baseline, serum osmolality did not differ from baseline in either trial (*P* ≥ 0.776). In HYP, urine osmolality increased from baseline to immediately post-exercise (*P* = 0.002) and remained elevated from baseline at 30 min post-exercise and 24 h post-baseline (*P* ≤ 0.002). In EU, urine osmolality did not change from baseline to immediately post-exercise (*P* = 0.744) or 30 min post-exercise (*P* = 0.072) but was elevated compared to baseline at 24 h post-baseline (*P* = 0.006). Osmolality of urine produced from leaving the laboratory on day 1 until returning the next day, was not different between trials (HYP: 580 ± 280 mOsm/kgH_2_O, EU: 450 ± 190 mOsm/kgH_2_O; *P* = 0.090). Total 24 h urine volume was not different between trials (HYP: 1745 ± 875 mL, EU: 2195 ± 990 mL; *P* = 0.079).

### Biomarkers of renal injury

**Fig. 2:**
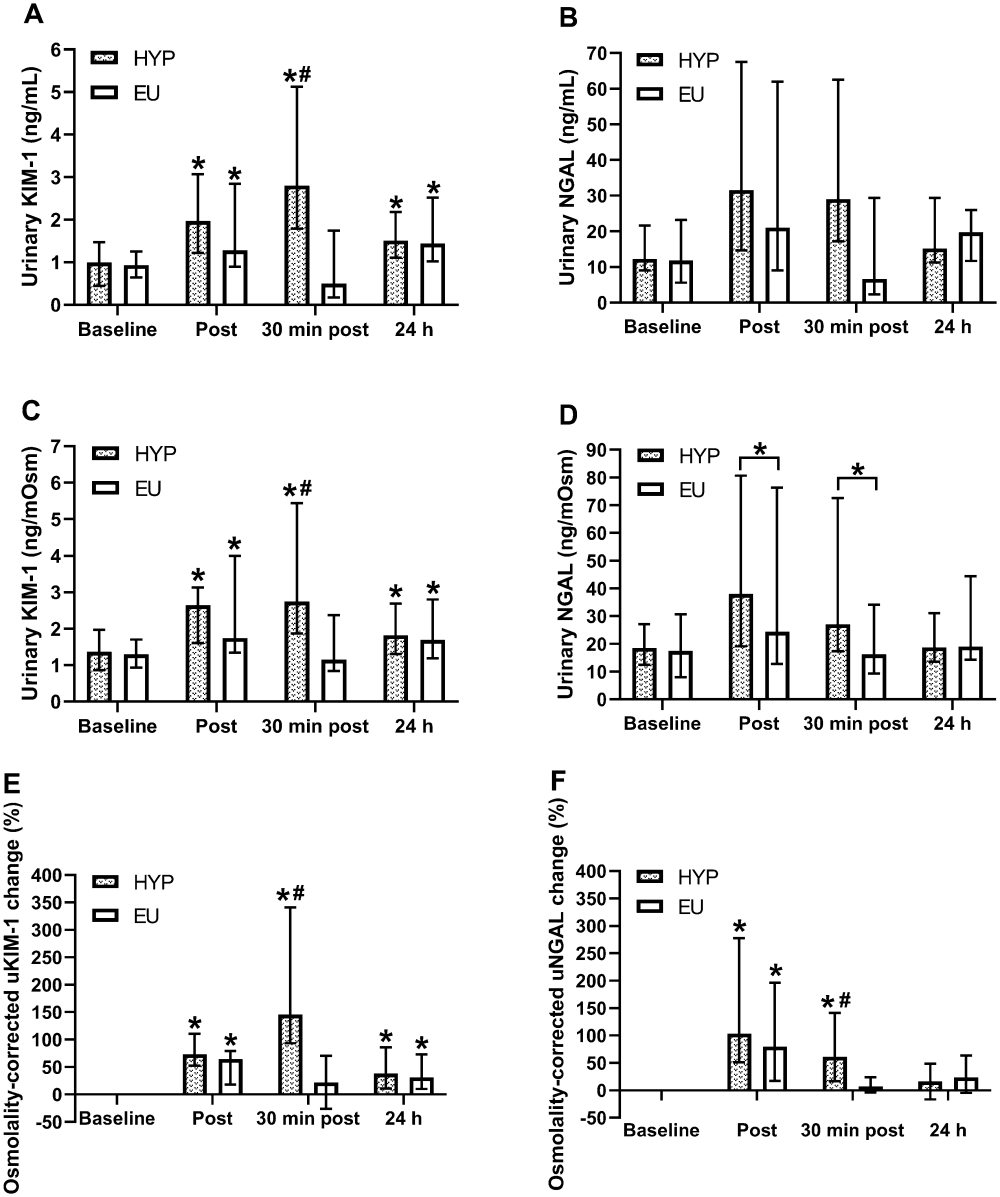
Urinary KIM-1 (**A**) and NGAL (**B**) concentrations, osmolality-corrected urinary KIM-1 (**C**) and NGAL (**D**) concentrations, and percentage change in osmolality-corrected urinary KIM-1 (**E**) and NGAL (**F**) concentrations. Timepoints include: baseline, immediately after exercise (post), 30 min after exercise (30 min post) and 24 h post-baseline (24 h). ^*^Indicates a significant difference from baseline; ^#^Indicates a significant difference between hypohydrated (HYP) and euhydrated (EU) trials. Data are presented as median with interquartile range

There was a trial by time interaction effect (*P* = 0.023: $\eta_p^2$= 0.318) for uKIM-1 concentrations (Fig. 2A), with 30 min post-exercise concentrations greater in HYP than EU (*P* = 0.012). In HYP, uKIM-1 concentrations increased from baseline to immediately post-exercise (*P* < 0.001), remaining elevated from baseline at 30 min post-exercise and 24 h post-baseline (*P* = 0.002). In EU, uKIM-1 concentrations increased from baseline to immediately post-exercise (*P* = 0.01), recovered to baseline levels at 30 min post-exercise (*P* = 0.730), but were elevated compared to baseline at 24 h post-baseline (*P* = 0.006). Correcting uKIM-1 concentrations for urine osmolality ($\eta_p^2$= 0.308; Fig. 2C) did not alter the significance of any of these findings, with 30 min post-exercise concentrations 138% higher in HYP than EU (*P* = 0.024). Expressing osmolality-corrected uKIM-1 concentrations as percentage changes from baseline (Fig. 2E) also did not alter the significance of any of these findings. There was no trial effect (*P* = 0.559), time effect (*P* = 0.057) or trial by time interaction effect (*P* = 0.185; $\eta_p^2$= 0.127) for uNGAL concentrations (Fig. 2B). Correcting uNGAL concentrations for urine osmolality (Fig. 2D) created a time effect (*P* = 0.041), with concentrations increasing from baseline to immediately post-exercise (*P* < 0.001), remaining elevated at 30 min post-exercise (*P* < 0.001), but recovering to baseline levels at 24 h post-baseline (*P* = 0.072). When osmolality-corrected uNGAL concentrations were expressed as percentage changes from baseline (Fig. 2F), there was a trial by time interaction effect (*P* = 0.009) with the percentage increase at 30 min post-exercise greater in HYP than EU (*P* = 0.033).

**Fig. 3:**
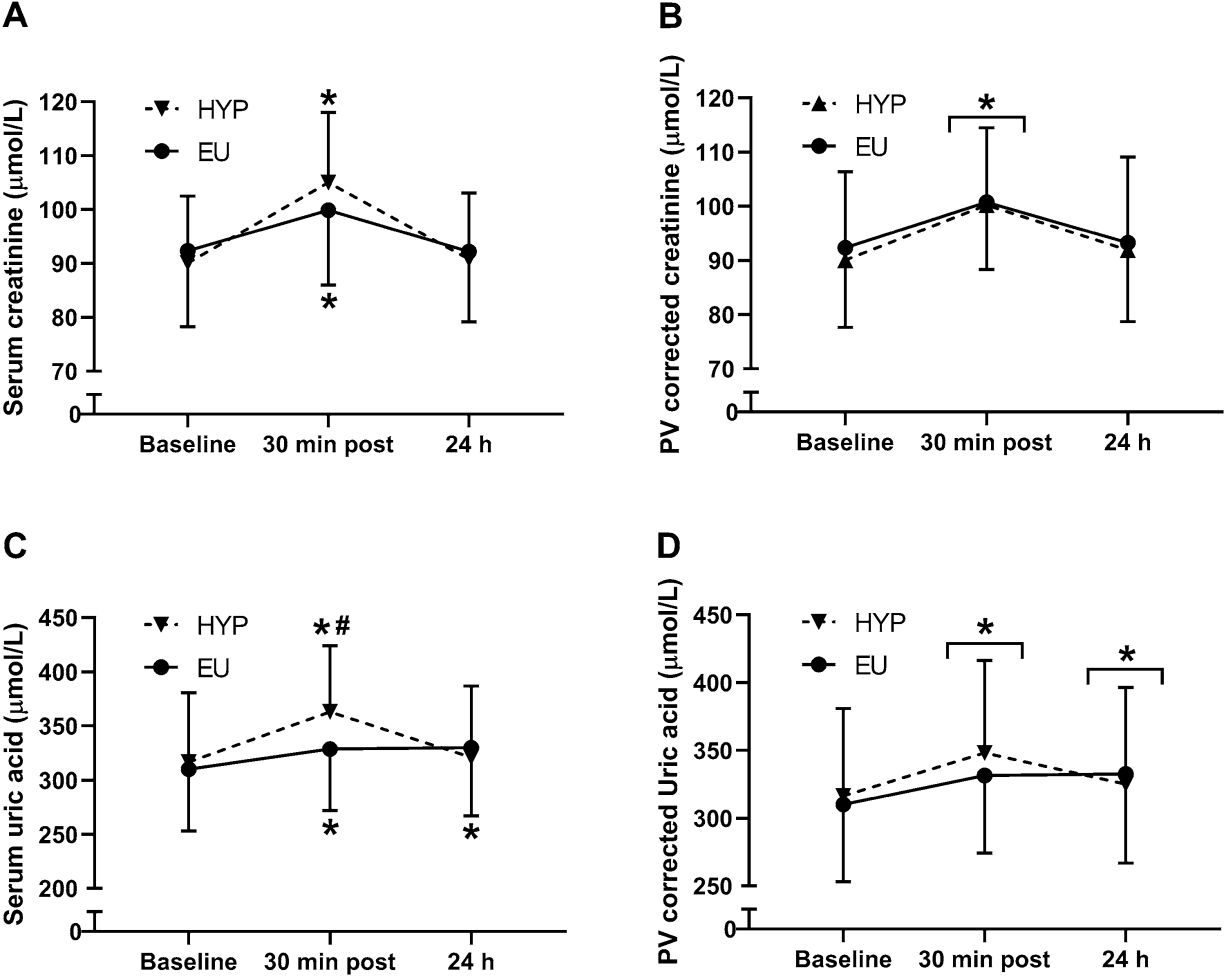
Serum creatinine (**A**), serum creatinine corrected for changes in plasma volume (**B**), serum uric acid (**C**) and serum uric acid corrected for changes in plasma volume (**D**) at baseline, 30 min after exercise (30 min post) and 24 h post-baseline (24 h). *Indicates a significant difference from baseline; ^#^Indicates a significant difference between hypohydrated (HYP) and euhydrated (EU) trials. Data are presented as mean ± standard deviation

There were trial by time interaction effects (*P* < 0.001) for serum creatinine ($\eta_p^2$= 0.510; Fig. 3A) and serum uric acid ($\eta_p^2$ = 0.620; Fig. 3C) concentrations, with both markers increasing from baseline to post-exercise in both trials (*P* ≤ 0.004). Post-exercise, serum uric acid concentrations were greater in HYP than EU (*P* < 0.001), but serum creatinine concentrations were not different between trials (*P* = 0.093). Correcting creatinine ($\eta_p^2$= 0.043; Fig. 3B) and uric acid ($\eta_p^2$ = 0.252; Fig. 3D) concentrations for changes in plasma volume removed the trial by time interaction effects (*P* ≥ 0.052), but the time effects remained (*P* ≤ 0.004). Plasma volume corrected creatinine concentrations increased from baseline to post-exercise (*P* < 0.001) and returned to baseline levels at 24 h post-baseline (*P* = 0.276), whereas plasma volume corrected uric acid concentrations increased from baseline to post-exercise (*P* < 0.001) and remained elevated at 24 h post-baseline (*P* = 0.040). When the effects of trial order were investigated, there were no trial by time interaction effects (*P* ≥ 0.207) or trial effects (*P* ≥ 0.238) for any biomarker of renal injury measured.

### Muscle damage

**Fig. 4:**
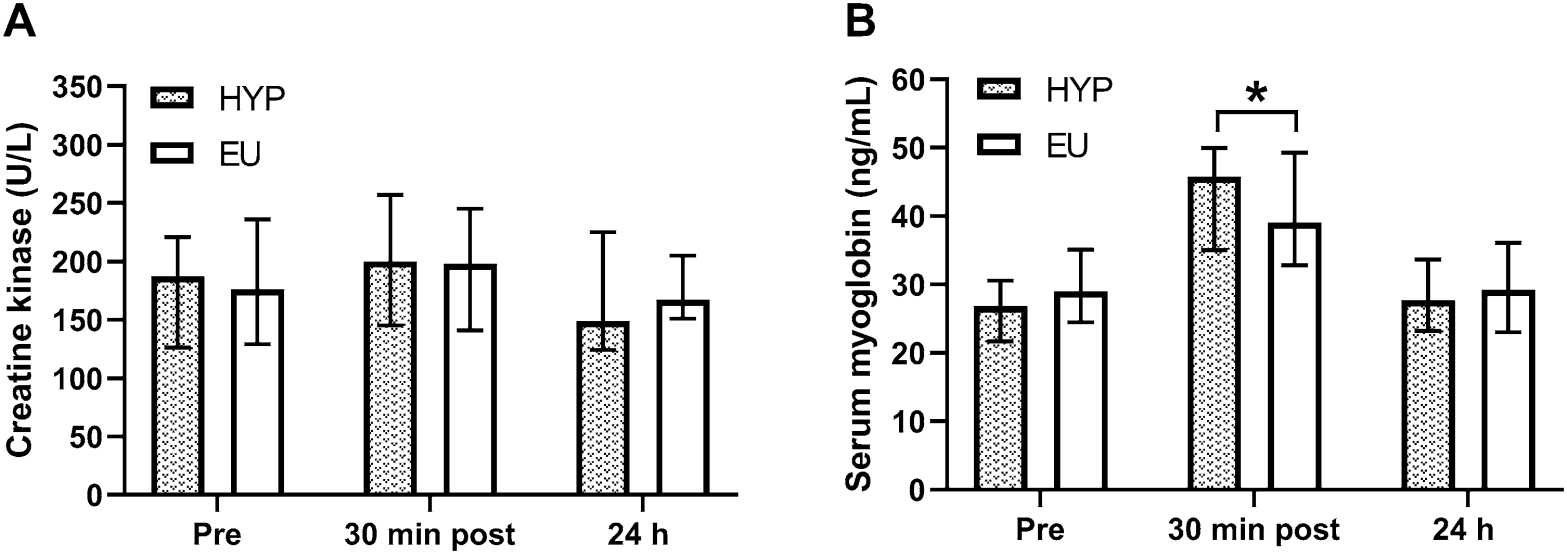
Serum creatine kinase (**A**) and serum myoglobin (**B**) concentrations at baseline, 30 min after exercise (30 min post) and 24 h post-baseline (24 h). *Indicates a significant difference from baseline within trial. Data are presented as median with interquartile range

There were no trial by time interaction effects (*P* ≥ 0.392) for serum CK ($\eta_p^2$= 0.063; Fig. 4A) or serum myoglobin ($\eta_p^2$= 0.016; Fig. 4B) concentrations. There was no time effect for serum CK concentrations (*P* = 0.108) but there was for serum myoglobin concentrations (*P* < 0.001), with concentrations increasing from baseline to post-exercise (*P* < 0.001) and returning to baseline levels at 24 h (*P* = 0.055). Correcting serum CK and myoglobin concentrations for changes in plasma volume did not alter the significance of any results, and thus these data are not presented. When assessing the effect of trial order on serum CK and myoglobin concentrations, there were no effects of trial (*P* ≥ 0.546) or trial by time interaction effects (*P* ≥ 0.747).

### Physiological response to exercise and perceptual measures

Average exercising HR (HYP: 153 ± 10 beats/min; EU: 148 ± 11 beats/min; *P* = 0.038) and RPE (HYP: 14 [13–15]; EU: 14 [12–14]; *P* = 0.017) were greater in HYP than EU. There was a trial by time interaction effect (*P* < 0.001) for thirst (Table 1), with thirst scores higher in HYP than EU 30 and 60 min into exercise, as well as at the end of exercise and post-exercise (*P* ≤ 0.012). There were no trial by time interaction effects (*P* ≥ 0.082) for headache, nausea, dizziness, GI bloating, GI comfort, stomach fullness, or urge to vomit, and despite a trial by time interaction effect (*P* = 0.009) for thermal sensation (Table 1) post hoc analysis indicated no significant difference between trials at any time point (*P* ≥ 0.126).

**Table 1:** Subjective feelings questionnaires scores at baseline, 30 min into exercise (30 min), 60 min into exercise (60 min), at the end of exercise (end), 15 min after exercise (post) and 24 h post-baseline (24 h)

	HYP						EU					
	Baseline	30 min	60 min	End	Post	24 h	Baseline	30 min	60 min	End	Post	24 h
Thirst (0–10)	4 [3–5]	5 [4–5] #	6 [6–7] **#**	9 [9–9] **#**	9 [8–9] **#**	4 [3–5]	4 [2–5]	3 [2–3]	3 [1–4]	2 [1–3]	2 [1–2]	4 [3–5]
Headache (0–10)	0 [0–0]	0 [0–0]	0 [0–1]	3 [0–4]	0 [0–1]	0 [0–0]	0 [0–0]	0 [0–0]	0 [0–0]	0 [0–1]	0 [0–0]	0 [0–0]
Nausea (0–10)	0 [0–0]	0 [0–0]	0 [0–0]	1 [0–2]	0 [0–0]	0 [0–0]	0 [0–0]	0 [0–0]	0 [0–0]	0 [0–0]	0 [0–0]	0 [0–0]
Dizziness (0–10)	0 [0–0]	0 [0–0]	0 [0–2]	2 [1–2]	0 [0–1]	0 [0–0]	0 [0–0]	0 [0–0]	0 [0–1]	1 [0–2]	0 [0–0]	0 [0–0]
GI bloating (0–10)	0 [0–0]	1 [0–2]	0 [0–2]	1 [0–1]	0 [0–0]	0 [0–0]	0 [0–1]	0 [0–1]	1 [0–2]	1 [0–4]	1 [0–3]	0 [0–0]
GI comfort (0–10)	0 [0–1]	0 [0–0]	0 [0–0]	0 [0–1]	0 [0–0]	0 [0–0]	0 [0–1]	0 [0–0]	0 [0–1]	1 [0–2]	0 [0–1]	0 [0–0]
Stomach fullness (0–10)	2 [1–2]	5 [3–5]	3 [1–4]	2 [1–3]	2 [1–3]	2 [1–2]	2 [1–3]	4 [3–5]	3 [2–5]	3 [2–5]	3 [1–4]	2 [0–2]
Urge to vomit (0–10)	0 [0–0]	0 [0–0]	0 [0–0]	0 [0–2]	0 [0–0]	0 [0–0]	0 [0–0]	0 [0–0]	0 [0–0]	0 [0–0]	0 [0–0]	0 [0–0]
Thermal sensation (-10–10)	0 [0–0]	3 [3–4]	4 [4–5]	6 [5–7]	0 [0–1]	0 [0–0]	0 [0–0]	4 [3–4]	4 [4–5]	5 [3–5]	0 [0–1]	0 [0–0]

^#^Indicates a significant difference between hypohydrated (HYP) and euhydrated (EU) trials. Data are presented as median [interquartile range]

### Food and fluid intake

From when the subjects left the laboratory on day 1 until they returned the next day, energy (HYP: 11,428 ± 3313 kJ, EU: 11,420 ± 3357 kJ; *P* = 0.989), carbohydrate (HYP: 354 ± 137 g, EU: 354 ± 130 g; *P* = 0.991), protein (HYP: 101 [92–118] g, EU: 103 [89–125] g; *P* = 0.894), fat (HYP: 95 ± 47 g, EU: 94 ± 43 g; *P* = 0.908) and sodium (HYP: 2680 ± 1097 mg, EU: 2622 ± 1499 mg; *P* = 0.847) intake were not different between trials. In this period, water intake (HYP: 3823 ± 841 mL, EU: 2990 ± 1003 mL; *P* = 0.006) and water from drinks (HYP: 2974 ± 763 mL, EU: 2174 ± 939 mL; *P* = 0.005) were greater in HYP than EU, whereas water from foods (HYP: 849 ± 316 mL, EU: 816 ± 370 mL; *P* = 0.491) was not different between trials. Total water intake for the duration of the trial (including pre-exercise and during exercise) was higher in EU than HYP (HYP: 4185 ± 829 mL, EU: 5360 ± 898 mL; *P* < 0.001).

## Discussion

The present study aimed to investigate the effect of manipulating hydration status during cycling in the heat on biomarkers of renal injury. The main findings were that osmolality-corrected uKIM-1 and uNGAL concentrations increased immediately after exercise, and that osmolality-corrected uNGAL (when expressed as percentage changes from baseline) and uKIM-1 concentrations were significantly greater 30 min after exercise when subjects were hypohydrated (compared to when they were euhydrated). These findings suggest that moderate intensity cycling in the heat increases renal injury, and the extent of renal injury is exacerbated by hypohydration.

The present study corrected the concentrations of uKIM-1 and uNGAL for urine osmolality, to account for the urine concentrating effect of hypohydration. The greater concentrations of osmolality-corrected uNGAL (when expressed as percentage changes from baseline) and uKIM-1 at 30 min post-exercise in HYP compared to EU, despite correction for urine osmolality, suggests that the production of these biomarkers was increased (rather than just increased urine concentration), and thus renal injury was increased in HYP. The expression of KIM-1 is increased in response to proximal tubular injury (Ichimura et al. 1998; Han et al. 2002; Kashani et al. 2017), whereas a rise in uNGAL is thought to be mainly due to an increase in synthesis from the distal nephron (Paragas et al. 2011; Helanova et al. 2014; Bongers et al. 2017, 2018). However, as a decrease in proximal tubular reabsorption has the potential to contribute to an increase in uNGAL concentrations, it is not certain that HYP increased injury to the distal nephron in the present study. Plasma/ serum NGAL concentrations were not measured in the present study as these are thought to be more indicative of renal blood flow than actual renal tubular injury, whereas uKIM-1 and uNGAL concentrations are thought to indicate renal tubular injury (Schaub and Parikh 2016; Schlader et al. 2019). Therefore, in agreement with the prior research by Juett et al. (2021b) and Chapman et al. (2020), the results from the present study suggest that hypohydration produced by cycling in the heat increased proximal tubular injury (compared to when euhydration was maintained with water ingestion) (Chapman et al. 2020; Juett et al. 2021b).

However, the results of the present study contrast the recent research by Haroutounian et al. (2021), who found no difference in uNGAL or uKIM-1 concentrations between a hypohydrated and euhydrated group after 90 min cycling (Haroutounian et al. 2021). There are a variety of potential explanations for the contrasting findings, including the shorter duration of exercise (90 min vs ~ 120 min), and thus lower level of hypohydration (2.4% vs 2.8%), and less time spent in a hypohydrated state, as well as the reduced statistical power of an independent group design (with the same number of total subjects) in Haroutounian et al. (2021) compared to the present study (Haroutounian et al. 2021). The most likely explanation for the contrasting findings, though, is the difference in urine sample timing (urine samples were taken immediately post-exercise in Haroutounian et al. 2021), which raises an important methodological consideration for future studies. Except in those that could not produce a void, we collected urine samples both immediately and 30 min post-exercise. The urine samples collected at 30 min post-exercise were more reflective of the difference in hydration status between trials, as all of this urine was produced at the time when subjects differed most in their hydration status, demonstrated by the significant difference in urine osmolality between HYP and EU trials 30 min post-exercise. The urine samples taken immediately post-exercise were reflective of all urine produced since the sample prior to exercise, and therefore values are an average of a euhydrated start point to a gradually accrued state of hypohydration. Interestingly, the immediately post-exercise urine samples in the present study produced similar results to Haroutounian et al. (2021), with no differences in urine osmolality, uKIM-1 or uNGAL concentrations between HYP and EU trials. Therefore, these results emphasise the importance of the timing of urine samples.

The increases in osmolality-corrected uKIM-1 and uNGAL concentrations immediately post-exercise, regardless of hydration status, suggest that cycling in the heat itself increased renal injury. This was likely via a reduction in renal blood flow due to the increased demands for skin and skeletal muscle blood flow, leading to renal ischaemia and subsequent renal injury (Radigan and Robinson 1949; Smith et al. 1952; Rowell 1974; Poortmans 1984; Basile et al. 2012). The greater concentrations of osmolality-corrected uNGAL (when expressed as percentage changes from baseline) and uKIM-1, in HYP compared to EU at 30 min post-exercise, suggested that hypohydration exacerbated this renal injury, potentially via the reduction in plasma volume and/or the increase in serum osmolality. A decrease in plasma volume can increase the activation of the renin–angiotensin–aldosterone system (RAAS), which may increase renal injury via a reduction in renal blood flow (Basile et al. 2012; Petejova and Martinek 2014; Cheuvront and Kenefick 2014; Schlader et al. 2019). The reduction in plasma volume in the hypohydrated trial of the present study was only ~ 4.5%, though. Therefore, whilst RAAS activation was not directly measured in the present study, this decrease in plasma volume is less than the threshold of an ~ 10% decrease in blood volume that is believed to be required to increase the activation of the RAAS (Cheuvront and Kenefick 2014). Consequently, this mechanism seems unlikely to have been responsible for the increased renal injury in HYP. Therefore, the increase in serum osmolality was the more likely mechanism via which hypohydration increased renal injury. An increase in serum osmolality of approximately 5/6 mOsm/kgH_2_O can result in an increase in arginine vasopressin (AVP) secretion (Cheuvront and Kenefick 2014; James et al. 2019). This may increase renal oxygen consumption and decrease renal blood flow, resulting in renal ischaemia and subsequent renal injury (Bragadottir et al. 2009; Basile et al. 2012). Whilst it is a limitation of the present study that neither AVP nor copeptin (a stable surrogate marker of AVP) were measured, it is thought that serum osmolality is the main regulator of circulating AVP concentrations during exercise (Wade 1984), something that we have previously reported with cycling exercise in the heat (James et al. 2017).

The hydration protocol used in the euhydrated trial of the present study (100% of body mass losses replaced with water ingestion) was well-tolerated, as evidenced by the lack of differences between HYP and EU for all subjective feelings questionnaires (except thirst), and also resulted in reductions in exercising HR and RPE. This hydration protocol was an aggressive strategy, designed to further the understanding of the effects of manipulating hydration status by maximising the difference between HYP and EU trials. However, the ingestion of such large volumes of plain water can increase the risk of overhydration and exercise-associated hyponatremia (Rosner and Kirven 2007) whilst it is also not commonly practiced by exercisers ((Mears and Shirreffs 2014)). Indeed, in the current study, there was a significant decrease in serum osmolality in EU. Therefore, future research should examine the effect of more commonly practiced (and less aggressive) hydration strategies during exercise on biomarkers of renal injury, such as preventing a > 2% body mass loss (Sawka et al. 2007). This strategy would still likely attenuate a rise in serum osmolality and subsequent AVP secretion (Cheuvront and Kenefick 2014), thereby reducing an important mechanism that is likely involved in renal injury (Bragadottir et al. 2009; García-Arroyo et al. 2017; Roncal-Jimenez et al. 2017; Mansour et al. 2019), while also minimising performance decrements (James et al. 2017, 2019; Funnell et al. 2019), but with a lower risk of exercise-associated hyponatremia.

At 24 h post-baseline in the present study, body mass was significantly lower, whereas urine osmolality and osmolality-corrected uKIM-1 concentrations were significantly higher, compared to baseline in both trials. Athletes will often exercise on consecutive days, and therefore starting with increased indicators of renal injury on a consecutive day of exercise could be of concern. It appears that only two studies have assessed the effects of repeated bouts of exercise on uNGAL and uKIM-1 concentrations, finding no cumulative effects (Bongers et al. 2017; Haroutounian et al. 2021). However, the effects of longer bouts of exercise than seen in Haroutounian et al. (2021), performed at a higher exercise intensity than seen in Bongers et al. (2017), and/or bouts of exercise that produce greater hypohydration than seen in these studies, is unknown and thus warrants investigation.

Cycling was chosen as the mode of exercise in the present study to minimise the effect of muscle damage on biomarkers of renal injury, whilst still allowing the findings to be applied to an exercising context. Whilst serum myoglobin concentrations did increase from pre- to post-exercise, they increased much less than in our previous study involving high intensity intermittent running exercise (Juett et al. 2021b). Furthermore, serum creatine kinase concentrations did not increase from pre- to post-exercise in the present study. Taken together, these findings suggest that the cycling exercise in the present study produced minimal muscle damage. However, to further isolate the effects of manipulating hydration status, in the complete absence of muscle damage, passive heating trials could have been done, with the same increase in core temperature. It is a limitation of the present study that core body temperature was not measured (due to the budget constraints of using telemetry pills and difficulties in recruitment with using rectal thermistors), as this would have allowed measurement of thermal strain and provided further understanding of the mechanisms involved. From a similar perspective there is debate surrounding the method of prescribing exercise intensity. Subjects completed two trials at 50% W_peak_ to replicate absolute intensity between trials where there would have been greater thermal strain in HYP. Not using fixed metabolic heat production to determine intensity may be considered a criticism, but this would have reduced the absolute workload in HYP and therefore comparison between trials would have been different. Both approaches have merit but it was decided a matched intensity commonly reflects real world practices where a fixed workload is initially set.

The results provide some interesting practical considerations for exercisers cycling in the heat, suggesting that water should be consumed during exercise to attenuate increases in biomarkers of renal injury. However, as mentioned previously, the fairly aggressive strategy employed in this study may increase the risk of exercise associated hyponatremia. Therefore, further study is needed to see if more typical and less aggressive voluntary fluid intake strategies attenuate biomarkers to the same extent. In the meantime, it may be prudent for those engaging in cycling in the heat to consume water to minimise hypohydration to < 2–3% body mass as this has been shown to improve cycling performance (Funnell et al. 2019) and may also attenuate rises in biomarkers of renal injury. In conclusion, the findings from the present study indicate that cycling in the heat increases renal injury, and that hypohydration further exacerbates proximal tubular injury (compared to when euhydration is maintained with water ingestion). This exacerbation may be mediated by serum hyperosmolality and subsequent AVP release. Future research should examine the effect of manipulating hydration status during repeated bouts of exercise, that are of relatively high-intensity and long duration, on uNGAL and uKIM-1 concentrations. The hydration protocols used in future studies should be less aggressive than complete replacement of body mass losses with water ingestion., and the timing of urine samples should be carefully considered.

## Data Availability

The datasets generated during the current study are available from the corresponding author on reasonable request.

## Abbreviations

(u)IGFBP7: (Urinary) insulin-like growth factor-binding protein 7
(u)KIM-1: (Urinary) Kidney injury molecule-1
(u)NGAL: (Urinary) Neutrophil gelatinase-associated lipocalin
AKI: Acute kidney injury
ANOVA: Analysis of variance
ATP: Adenosine triphosphate
BMI: Body mass index
CK: Creatine Kinase
CKD: Chronic kidney disease
CV: Coefficient of variation
ELISA: Enzyme-linked immunosorbent assay
GI: Gastrointestinal
HR: Heart rate
Osm: Osmolality
RAAS: Renin–angiotensin–aldosterone system
RPE: Rating of perceived exertion
SD: Standard deviation
